# The impact of an innovative community-based peer-led intervention on uptake and coverage of sexual and reproductive health services among adolescents and young people 15–24 years old: results from the Yathu Yathu cluster randomised trial

**DOI:** 10.1186/s12889-024-18894-z

**Published:** 2024-05-28

**Authors:** Mwelwa Muleba Phiri, Albertus Schaap, Bernadette Hensen, Lucheka Sigande, Melvin Simuyaba, Lawrence Mwenge, Rosemary Zulu-Phiri, Louis Mwape, Sian Floyd, Sarah Fidler, Richard Hayes, Musonda Simwinga, Helen Ayles

**Affiliations:** 1 Zambart, Zambart House, UNZA Ridgeway Campus, off Nationalist Road, Lusaka, Zambia; 2 Department of Infectious Disease Epidemiology, London School of Hygiene and Tropical Medicine, London, UK; 3 Sexual Health Unit, Institute of Tropical Medicine, Antwerp, Belgium; 4 Clinical Research Department, London School of Hygiene and Tropical Medicine, London, UK; 5 Imperial College and Imperial College NIHR BRC, London, UK

**Keywords:** Adolescents and young people, Sexual and reproductive health, HIV testing, Coverage, Implementation science

## Abstract

**Background:**

The Yathu Yathu (“For Us, By Us”) cluster-randomized trial (CRT) evaluated a peer-led community-based sexual and reproductive health(SRH) intervention implemented to address persistent barriers to SRH service use among adolescents and young people (AYP). We report the impact of the intervention on coverage of key SRH services among AYP.

**Methods:**

The trial was conducted from Jul 2019-Oct 2021 in two urban communities in Lusaka, Zambia, divided into 20 zones (~ 2350 AYP/zone). Zones were randomly allocated to intervention (*N* = 10) or control (*N* = 10) arm. In all zones, a census was conducted and all AYP aged 15-24-years offered participation. The intervention consisted of peer-led community-based hubs providing SRH services; a prevention points card (PPC) system to incentivize and track SRH service use and community engagement. This paper reports on the outcome of coverage (accessing at least one key SRH service), comparing intervention and control arms using PPC data and standard methods of analysis for CRTs.

**Results:**

Among enumerated AYP, 93.6% (14,872/15,894) consented to participate from intervention zones and 95.1% (14,500/15,255) from control zones. Among those who accepted a PPC, 63.8% (9,493/14,872) accessed at least one key SRH service during the study period in the intervention arm, compared to 5.4% (776/14,500) in the control arm (adjPR 12.3 95%CI 9.3–16.2, *p* < 0.001).

**Conclusions:**

The Yathu Yathu intervention increased coverage of key SRH services among AYP and reached two-thirds of AYP. These findings demonstrate the potential of providing peer-led community-based SRH services.

**Trial registration:**

ISRCTN75609016 (11/10/2021), clinicaltrials.gov number NCT04060420 (19/08/2019); retrospectively registered.

**Supplementary Information:**

The online version contains supplementary material available at 10.1186/s12889-024-18894-z.

## Background

In 2021, the number of new HIV infections in Eastern and Southern Africa was ˜670,000. New infections among adolescent girls and young women aged 15–24 (AGYW) accounted for 25% of these and infections among adolescent boys and young men of the same age (ABYM) contributing 8% [1]. According to the 2016 Zambian Population-based HIV Impact Assessment (ZAMPHIA), 32% of adolescents and young people aged 15–24 years (AYP) had tested for HIV recently (in the last 12 months) [2]. Among AGYW aged 15–24 years, 40% self-reported recent HIV testing compared to 25% of ABYM [2].

The high HIV burden and morbidity associated with HIV, unintended pregnancies, and sexually transmitted infections (STI) show that improved sexual and reproductive health (SRH) strategies, including increasing contraceptive use among AGYW are needed [3, 4]. According to the World Health Organization, five key dimensions should be considered to address SRH needs of AYP:, namely services should be equitable, accessible, acceptable, appropriate and effective [5]. These dimensions address barriers to SRH services such as lack of access to acceptable services, low sexual health knowledge, distance to facilities, restrictive laws and negative attitudes from healthcare staff [3, 6]. Therefore, delivery of acceptable SRH interventions for AYP requires concerted efforts from different actors involved in their implementation, including end-users.

Part of the UN Sustainable Development Goal 3 includes achieving universal coverage of SRH services by 2030 [7]. However, with persisting barriers to SRH service access among AYP and gaps in coverage, achieving universal coverage requires new initiatives. While there is evidence of positive effects for interventions that are a combination of healthcare worker training, adolescent friendly facility improvements, such as youth friendly corners, and information provision, there is need for further, rigorous evidence on community-based interventions and their impact on coverage [8, 9]. Evidence from the HPTN 071(PopART) trial, a large community-randomised trial of universal HIV testing and treatment in Zambia and South Africa, showed that community-based services reached AYP and were acceptable to them, but challenges remained in maintaining coverage among AYP with persistent gaps in reaching young men [10, 11].

The Yathu Yathu study evolved from lessons learnt from the HPTN 071 trial, and a consultative meeting with AYP who participated in this trial. The Yathu Yathu (“For us, by us”) intervention, co-developed with young people, healthcare workers, parents and guardians [12], was a community-based peer-led model of comprehensive SRH service delivery that used innovative “prevention points” cards (PPC) to incentivise service use with the cards intended to “nudge” participants towards using services [13]. The Yathu Yathu intervention, with an embedded process evaluation to optimise implementation, was implemented in two urban communities in Lusaka, Zambia, and evaluated through a cluster-randomised trial (CRT) [14].

The impact of the intervention on the primary outcome, knowledge of HIV status, has been reported previously [15] and showed higher knowledge of HIV status in the intervention arm compared to control (73.3% versus 48.4%, respectively, adjPR 1.53 95% CI 1.36, 1.72; *p* < 0.001). In this paper, we report the impact of Yathu Yathu on coverage of key SRH services (HIV testing, condom collection, contraceptive use, voluntary medical male circumcision (VMMC), pre-exposure prophylaxis (PrEP) initiation and anti-retroviral therapy (ART) initiation), and an evaluation of intervention implementation.

## Methods

Trial findings are reported in line with CONSORT 2010 extended guidelines for CRTs.

### Study design and participants

Yathu Yathu (“for us, by us”) was a two-arm CRT conducted in two densely populated urban communities in Lusaka, Zambia, from July 2019 to October 2021 with intervention implementation from September 2019-September 2021. Each community was split into 10 geographical zones(clusters), with each zone having a population of ˜2350 AYP.

### Randomisation and masking

In a public ceremony with study community members, these 20 zones were randomly allocated to intervention or control, with 5 intervention and 5 control zones in each community [14]. Restricted randomization was used to ensure the two trial arms were balanced on: participation in the PopART intervention during the last year (2017) of delivery, knowledge of HIV status among AYP who participated in the PopART intervention during its last year, uptake of HIV testing among AYP who participated in the PopART intervention during its last year, average population of AYP per zone, and distance from the centre of the zone to the local health facility [14].

During the first five months of the study, enumerators went door-to-door and enumerated all household members. All AYP present in the household were invited to participate in the study. Repeated visits were conducted to include those not present during the initial visit. In both trial arms, those consenting to participate were given a Yathu Yathu PPC. The PPC was used to incentivize service uptake by allowing AYP to accrue points for accessing services and use these points to “purchase” rewards in the intervention and control arm; the PPC also allowed the study to monitor service use. The trial is described in detail elsewhere [14]. The process evaluation was guided by the Medical Research Council framework for process evaluations of complex interventions [16].

### Description of the intervention

The intervention consisted of three main components: the provision of SRH services in community-based hubs (one hub per intervention zone) located away from, but linked to, the main healthcare facility (one per community), the prevention points system, and community engagement in the ten zones randomized to receive the intervention. SRH services, including HIV testing, STI screening, comprehensive sexuality education (CSE) sessions, edutainment sessions, condom demonstrations and provision, were provided at the hubs by peer support workers (PSWs), supervisors and nurses. We defined “peer” as someone with whom one shares demographic or social similarities. This is because our recruitment of peers was not only based on age but also on social similarities, such as the community the peer resided in (we recruited staff that lived in the communities that they would work in). It also included consideration of their qualifications and work experience. These considerations were driven by learnings from AYP themselves during the formative study to finalise the design of the intervention [17]. Hubs were in central areas of the intervention zone and had a minimum of two separate rooms. At each of the two health facilities, an information desk facilitated linkage to care (for VMMC, ART, long-acting contraception, PrEP and PEP) and helped AYP navigate the health facility.

AYP from the intervention zones could gain points for accessing SRH services at the hub or at the local health facility. AYP from control zones could gain points for accessing SRH services from the local health facility only. If AYP from control zones attended a hub in an intervention zone they could access services but did not gain any points.

Community engagement was delivered in intervention zones, including one-on-one interactions, health talks, and door-to-door sensitization, regular meetings with existing adolescent community advisory boards (aCABs), adult community advisory boards (CABs) and parents/guardians throughout the implementation period. Community engagement in the control zones was restricted to the enumeration phase. Details of the intervention description using the TIDieR guidance can be found here: https://tidierguide.org/#/gen/5Vp65QYLv.

### Data collection at the hubs and clinic

We used electronic handheld devices to record the uptake of services and redemption of points for rewards by each participant by scanning the unique identifier (PPC Barcode). The identity of the participant was verified using a passport sized picture taken at enumeration.

### Outcomes

The impact of the intervention was measured by comparing the uptake of pre-defined key services between intervention and control arms. The key services were HIV testing, condom collection, hormonal contraceptive use, VMMC, PrEP initiation and ART initiation. The primary outcome (coverage) was defined as: uptake of one or more of the key services during the whole implementation period (September 2019-September 2021) as the numerator, among all AYP that consented (accepted a card) to participate as the denominator and among all AYP enumerated. A secondary analysis restricted this outcome to the last 12 months of implementation (October 2020-September 2021).

Guided by domains in the MRC Process Evaluation Framework, our implementation outcomes were: fidelity, defined as the consistency of what is implemented in practice with what was planned in terms of services available for access by AYP; reach, defined as the number of participants attending the hubs, by age and sex; dose-delivered, defined as the overall total number of services delivered, and dose-received, defined as the per capita number of services accessed by participants.

### Data analysis

Using enumeration data, we first described the number of households and household members approached, AYP present in the households and acceptance of the intervention (consenting and accepting a PPC) by arm, age and sex. We assessed whether trial arms were balanced in terms of AYP absent from the household at the time of enumeration. To assess factors associated with absence, we combined data from the intervention and control zones, as there was no difference in levels of absence across arms. We used logistic regression to investigate factors associated with absence, overall and separately for male and female AYP, with robust standard errors to allow for clustering within zones. To calculate adjusted odds ratios, we controlled for factors with weak evidence of association in unadjusted analysis *(p* < 0.1) using the Wald test.

For summaries on intervention reach, we described the proportion of AYP who received a PPC during enumeration and the numerator those who attended the hub and/or clinic at least once, for any service, disaggregated by arm, age and sex. Restricting analyses to AYP in the intervention arm, we assessed factors associated with accessing any service at the hub or clinic using logistic regression and considered age, sex, marital status, education, community, and household size as risk factors.

For dose-delivered, we described the total number of services delivered, disaggregated by arm, age and sex. Dose-received is expressed as the total number of services of a particular type accessed divided by the number of participants that accessed any service at the hub or clinic. We removed services considered to be duplicate, so services recorded as accessed on the same day, same location, same time and same participant (0.23%).

For the primary outcome analysis, we used standard methods of analysis for CRT with < 15 clusters/arm [18], with each zone being a cluster. We calculated coverage in each of the 20 zones and compared intervention with control zones by calculating a prevalence ratio (PR). The PR was calculated as the geometric mean of the prevalence of coverage across the 10 intervention zones, divided by the geometric mean of the prevalence of coverage across the 10 control zones.

In unadjusted analysis, we fitted a linear regression model with log (service coverage) as the outcome (20 values, one for each zone) and trial arm as the explanatory variable. In adjusted analysis, a logistic regression model was fitted to the individual-level data on each study participant, using sex, age, and community (stage 1) as explanatory variables to predict the probability of accessing at least one service under the null hypothesis of no intervention effect. For cluster-level analysis (stage 2), for each zone we calculated the ratio of observed(O) to expected(E) number of individuals who accessed at least one service, and then calculated the log (ratio-residual) as log (O/E). We then fitted a linear regression model of log (O/E) on trial arm to obtain the adjusted prevalence ratio comparing intervention zones with control zones. For age/sex subgroup and for each SRH service analysis, the above analysis was repeated, adjusting only for community at stage 1 of the analysis. At stage 2, if no individuals accessed services in a particular cluster (observed = ‘0’), we substituted the number observed with the value ‘1’ (to enable analysis of log(O/E) values. Analysis was conducted to include participation in the last 12 months as the initial statistical analysis plan was based on the anticipated duration of implementation. However, due to the COVID-19 pandemic and subsequent closure of hubs [19], the implementation period was extended by 9 months to cover this extension and is referred to as the “whole study period”.

#### Role of the funding source

The funders of the study had no role in study design, data collection, data analysis, data interpretation, or writing of the report. All authors had final responsibility for the decision to submit for publication.

## Results

**Fig. 1:**
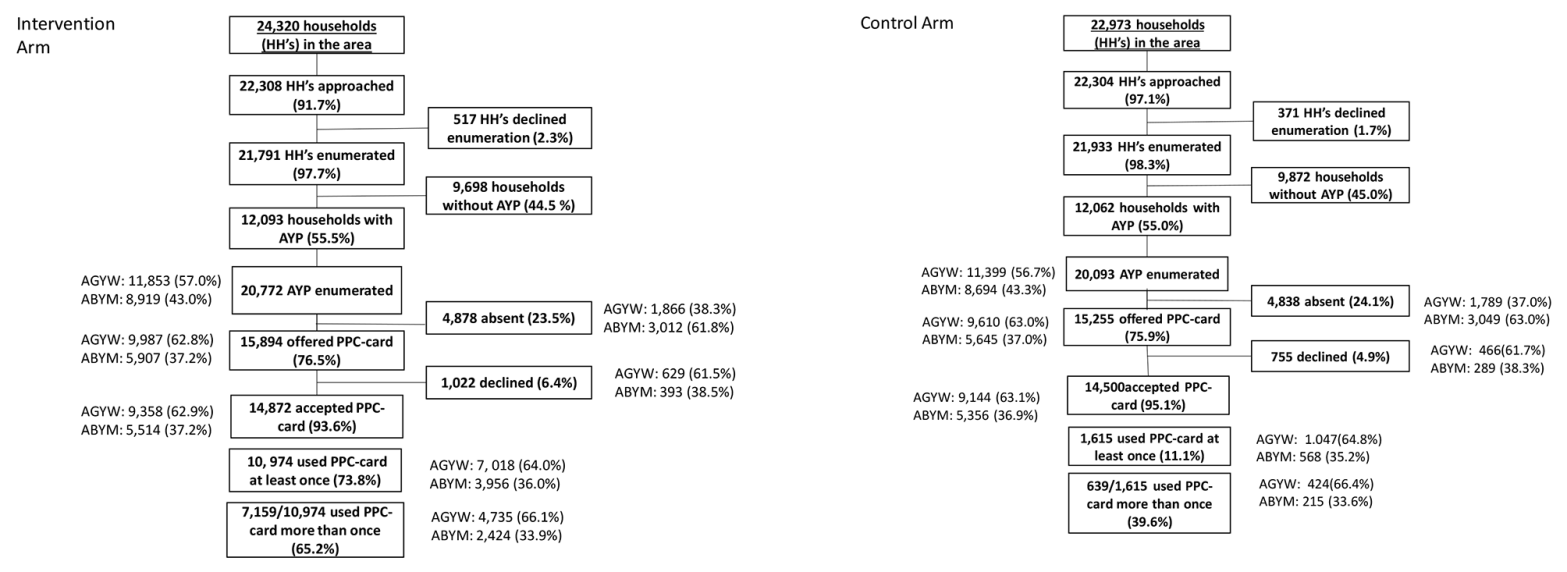
Flowchart of participation in the trial by study arm

Overall, 98.0% (43,724/44,612) of the households approached agreed to enumeration. Over half (24,155/43,724, 55.2%) had at least one household member aged between 15 and 24. Overall, 40,865 AYP were enumerated across the 20 zones, with more AGYW (23,252/40,865, 56.9%) enumerated than ABYM (17,613/40,865, 43.1%). Among enumerated AGYW, 19,597/23,252 (84.3%) were present in the household and offered a PPC. Among enumerated ABYM, 11,552/17,613 (65.6%) were present at the household and offered a PPC. Acceptance of the PPC was high among all present AYP (AGYW: 92.7%,18,502/19,597 and ABYM: 94.1%, 10,870/11,552), and similar across arms (Fig. 1).

Absence of AYP from home during enumeration was similar by arm (Fig. 1). More ABYM (34.4%; 6,061/17,613) were absent during enumeration than AGYW (15.7%; 3,655/23,252). Age, sex, education, marital status and the number of household members lived with were associated with being absent during enumeration (Table 1).

**Table 1:** Characteristics of adolescents and young people aged 15–24 enumerated and factors associated with absence during enumeration for the Yathu Yathu study

Adolescent girls and young women aged 15–24							Adolescent boys and young men aged 15–24					
	Characteristics of those enumerated	Number (row %) absent	Cluster-adjusted OR (95%CI)	*p*-value	*Adjusted OR (95%CI)	*p*-value	Characteristics of those enumerated	Number (row %) absent	Cluster-adjusted OR (95%CI)	*p*-value	**Adjusted OR (95%CI)	*p*-value
**Overall**	23,252 (100)	3655 (15.7)					17,613 (100)	6061 (34.4)				
**Arm**				0.98		0.87				0.659		0.659
Intervention	11,853 (51.0)	1866 (15.7)	1.00 (0.75–1.34)		0.98 (0.74–1.29)		8919 (50.6)	3012 (33.8)	0.94 (0.73–1.22)		0.95(0.74–1.21)	
Control	11,399 (49.0)	1789 (15.7)	1.00		1.00		8694 (49.4)	3049 (35.1)	1.00		1.00	
**Community**				0.191		0.289				0.224		0.243
Community 1	11,528 (49.6)	1953 (16.9)	1.00		1.00		9087 (51.6)	3279 (36.1)	1.00		1.00	
Community 2	11,724 (50.4)	1702 (14.5)	0.83 (0.63–1.10)		0.86 (0.66–1.13)		8526 (48.4)	2782 (32.6)	0.86 (0.67–1.10)		0.87 (0.68–1.10)	
**Age group**				< 0.001		**< 0.001**				< 0.001		**< 0.001**
15–17	6906 (29.7)	1485 (21.5)	1.76 (1.47–2.10)		1.06 (0.90–1.27)		5539 (31.4)	1811 (32.7)	0.79 (0.69–0.89)		0.81 (0.70–0.93)	
18–19	5101 (21.9)	653 (12.8)	0.94 (0.83–1.07)		0.63 (0.56–0.72)		4102 (23.3)	1202 (29.3)	0.67 (0.61–0.73)		0.69 (0.63, 0.77)	
20–24	11,245 (48.4)	1517 (13.5)	1.00		1.00		7977 (45.3)	3048 (38.2)	1.00		1.00	
**Educational Attainment**				0.001		**0.002**				0.137		0.015
No/primary	5193 (22.3)	692 (13.3)	0.78 (0.68–0.91)		0.80 (0.69–0.92)		3417 (19.4)	1234 (36.1)	1.10 (0.97–1.24)		1.16 (1.03–1.31)	
Secondary	18,059 (77.7)	2963 (16.4)	1.00		1.00		14,196 (80.6)	4827 (34.0)	1.00		1.00	
**Marital status**				< 0.001		**< 0.001**				< 0.001		**< 0.001**
Single/never married	17,559 (75.5)	3458 (19.7)	1		1.00		16,668 (94.6)	5551 (33.3)	1.00		1.00	
Married/cohabiting	5693 (24.5)	197 (3.5)	0.15 (0.12–0.18)		0.16 (0.14–0.19)		945 (5.4)	510 (54.0)	2.35 (1.70–3.25)		2.48 (1.72–3.58)	
**Number of HH members**				< 0.001		**0.001**				0.002		**< 0.001**
1–4	8727 (37.5)	866 (9.9)	1.00		1.00		5776 (32.8)	1815 (31.4)	1.00		1.00	
5–7	9533 (41.0)	1809 (19.0)	2.13 (1.93–2.35)		1.29 (1.12–1.47)		7476 (42.5)	2645 (35.4)	1.19 (1.06–1.35)		1.43 (1.27–1.61)	
> 7	4992 (21.5)	980 (19.6)	2.22 (1.89–2.60)		1.27 (1.05–1.53)		4361 (24.8)	1601 (36.7)	1.27 (1.11–1.44)		1.49 (1.31–1.69)	

*adjusted for community, age group, education, marital status and number of household (HH) members

**adjusted for community, age group, marital status and number of HH members

^1^ Age at time of consent to receive a Yathu Yathu prevention points card (PPC); OR = Odds ratio

### Fidelity

Almost all (95%) of the 40 staff members recruited and trained were retained, with hubs open for 452/532 (85%) potential working days. All but one, availability of books (13/14; 93%), of services intended to be provided at the hubs was provided (Table 2). In the hubs in both communities there were sporadic partial stock outs of oral contraceptives and HIV self-test kits. At facilities, there were sporadic partial stock outs of intra-uterine devices (IUD), contraceptive implants, oral contraceptives, and syphilis and HIV testing kits. COVID-19 related supply chain interruptions caused most stock-outs. In intervention zones, 816 community engagement activities were conducted compared to 771 planned activities due to COVID-19.

**Table 2:** Fidelity of the Yathu Yathu implementation of the intervention

Intended intervention content (services/activities)	Delivery of content (Y/*N*)	Deviations of delivery*
HIV counselling and HIVST (including secondary distribution of HIVST kits and support with couples-testing)	Y	Stock outs of HIVST
Information and referral for ART initiation	Y	
Demonstrations of how to put on a condom, provision of male and female condoms and lubricants	Y	
Information and advice on contraceptives and referral for contraceptive services, provision of emergency contraceptives and contraceptive pill	Y	Stock outs of oral and injectable contraceptives
Pregnancy testing, support to pregnant women (including phone-based support to HIV-positive) and encouragement to join ANC services at local health facilities	Y	
Information and referral for pre-exposure prophylaxis (PrEP) and post exposure prophylaxis (PEP)	Y	
Screening for TB and referral for diagnosis and treatment	Y	
Information and referral for VMMC services at the government health facility	Y	
information, screening and referral to the local health facility for diagnosis and treatment of STIs	Y	
Offer AYP points for accessing SRH services and allow AYP to redeem these points for rewards	Y	Points for activities and services were reviewed and changed after initial 5 month pilot phase.
Provide edutainment, including screening of videos with information on SRH	Y	Country wide load shedding of electricity and laptop breakdown affected frequency of provision
Refer AYP to other organisations providing vocational skills training, entrepreneurship	Y	
Referral individuals to gender-based violence (GBV) services	Y	
Have books available for AYP	N	

*All services were affected by COVID-19 from March 2020- September 2021

### Reach and factors associated with using PPC to access services at least once

Among all AYP who accepted a PPC in the intervention zones, 73.8% (10, 974/14,872) used their card at least once to access any service, with reach similar by sex: 75.0% (7,018/9,358) among AGYW and 71.7% (3,956/5,514) among ABYM (Table 3); 98.2% (10,780/10,974) only accessed services at hubs, 0.02% (2/ 10,974) only accessed services at the clinic, and 1.8% (194/10,974) accessed services at both. Among those that accessed at least once service, two-thirds (65.2%, 7,159/10,974) visited the hub more than once (Fig. 1). AGYW from community 2 were less likely to access services compared to AGYW from community 1 (69.3% vs. 81.2%) (aOR 0.54, 95%CI 0.35–0.82) with a similar effect among ABYM. Among all AYP, younger age groups were more likely to access services compared to AYP aged 20–24 (AGYW: aOR 2.39, 95%CI 1.96–2.90 aged 15–17; aOR 1.51, 95%CI 1.32–1.74 aged 18–19: ABYM: aOR 2.37, 95%CI 2.09–2.68 aged 15–17; aOR 1.61 95%CI 1.40–1.85 aged 18–19) (Table 3). AGYW living in households with > 4 household members were more likely to access services than those living in households with fewer members (5-7members: aOR 1.39, 95%CI 1.23–1.57; >7 members: aOR 1.54–2.21). For all AYP, no associations were found between education, marital status, and accessing services in intervention zones.

**Table 3:** Characteristics of adolescents and young people aged 15–24 using a PPC for any service and factors associated with using a PPC among those that accepted a PPC in the intervention arm

Adolescent girls and young women aged 15–24							Adolescent boys and young men aged 15–24					
	Characteristics of participants	Number (row %) using PPC	Cluster-adjusted OR (95%CI)	*p*-value	*Adjusted OR (95%CI)	*p*-value	Characteristics of participants	Number (row %) using PPC	Cluster-adjusted OR (95%CI)	*p*-value	**Adjusted OR (95%CI)	*p*-value
**Overall**	9358 (100%)	7018 (75.0)					5514 (100)	3956 (71.7)				
**Community**												
Community 1	4509(48.2)	3660(81.2)	1	0.003	1	**0.005**	2850(51.7)	2176(76.4)	1	0.007	1	**0.009**
Community 2	4849(51.8)	3358(69.3)	0.52(0.34,0.81)		0.54(0.35,0.82)		2664(48.3)	1780(66.8)	0.62(0.44–0.88)		0.64(0.45,0.89)	
**Age group**												
15–17	2967(31.7)	2524(85.1)	2.81(2.39,3.31)	< 0.001	2.39(1.96,2.90)	**< 0.001**	2016 (36.6)	1626 (80.7)	2.55 (2.19, 2.97)	< 0.001	2.37(2.09,2.68)	**< 0.001**
18–19	2032(21.7)	1575(77.5)	1.70(1.49,1.95)		1.51(1.32,1.74)		1341(24.3)	992(74.0)	1.74(1.50, 2.01)		1.61(1.40,1.85)	
20–24	4359(46.6)	2919(67.0)	1.00		1.00		2157(39.1)	1338(62.0)	1.00		1.00	
**Educational Attainment**												
No/primary	2185(23.4)	1682(77.0)	1.15(0.96,1.38)	0.126	1.02(0.89–1.18)	0.780	1065(19.3)	790(74.2)	1.16(1.02,1.33)	0.022	0.92(0.82,1.04)	0.183
Secondary	7173(76.7)	5336(74.4)	1.00		1.00		4449(80.7)	3166(71.2)	1.00		1.00	
**Marital status**												
Single/never married	6917(73.9)	5428(78.5)	1	< 0.001	1	0.509	5323(96.5)	3846(72.3)	1.00	0.003	1	0.342
Married/cohabiting	2441(26.1)	1590(65.1)	0.51(0.44,0.60)		0.95(0.82,1.10)		191(3.5)	110(57.6)	0.52(0.34,0.81)		0.85(0.60,1.19)	
**Number of HH members**												
1–4	3576(38.2)	2397(67.0)	1.00	< 0.001	1.00	**< 0.001**	1822(33.0)	1147(63.0)	1.00	< 0.001	1.00	**< 0.001**
5–7	3764(40.2)	2945(78.2)	1.77(1.58,1.98)		1.39(1.23,1.57)		2330(42.3)	1778(76.3)	1.90(1.63,2.21)		1.55(1.35,1.79)	
> 7	2018(21.6)	1676(83.1)	2.41(2.01,2.88)		1.84(1.54,2.21)		1362(24.7)	1031(75.7)	1.83(1.56,2.15)		1.58(1.36,1.83)	

*adjusted for community, age group, marital status and number of household (HH) members

**adjusted for community, age group, education, marital status and number of HH members

^1^ Age at time of consent to receive a Yathu Yathu prevention points card (PPC); OR = Odds ratio

Among all AYP who accepted a PPC in control zones, 11.1% (1,615/14,500) used their card at least once to access services, 90.3% (1,458/1,615) only accessed services at the clinic, 6.3% (101/,1,615) only accessed services at the hub and 3.5% (56/1,615) accessed services at both. Among those that accessed at least one service, less than half (39.2%, 639/1,615) visited the hub or clinic more than once.

### Dose of services delivered and received

A total of 158,856 services were delivered and received by 10,974 participants during 65,521 visits during the implementation period in the intervention arm. A total of 5,309 services were delivered and received by 1,615 participants during 3,546 visits in the control arm. Details of per capita service delivery by arm, age, sex and service are presented in the Supplementary Appendix.

**Fig. 2:**
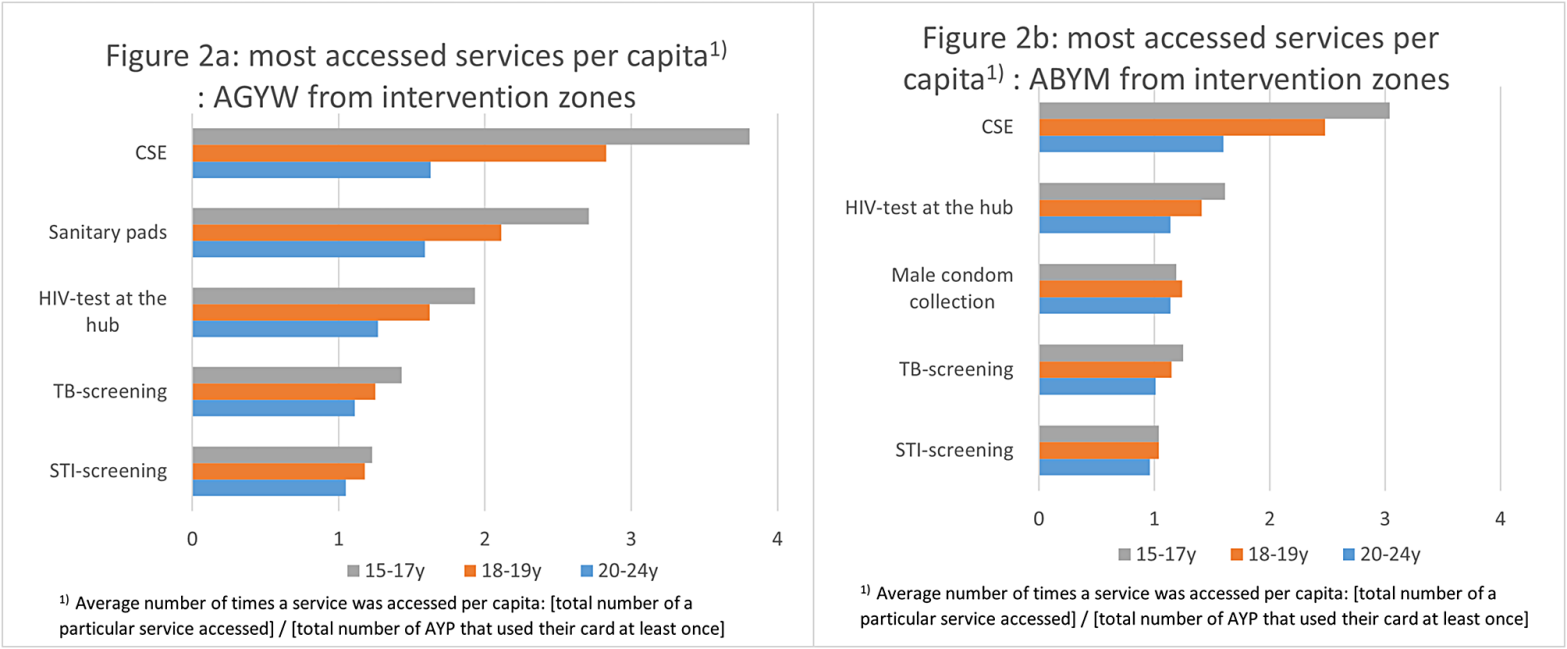
Uptake of services in intervention zones (top five services only) by age and sex

Among AGYW, CSE was the most accessed service and accessed on average 3.8 times by AYP aged 15-17years, 2.8 times by AYP aged 18–19-years and 1.6 times by AYP aged 20–24-years. The second most popular services were collection of menstrual pads and HIV testing at the hubs (Fig. 2a). Among ABYM, CSE was the most accessed service, accessed on average 3.0 times by AYP aged 15-17years, 2.5 times by AYP aged 18-19years and 1.6 times by AYP aged 20–24-years. The second most popular services were HIV testing at the hubs, male condom collection and TB screening (Fig. 2b). Among all AYP, the least accessed services were TB treatment initiation, collection of PrEP and initiation of PEP.

### Prevention points system: points gained and redeemed

In total 13,840,880 points were gained by AYP from intervention zones, with 91.9% (12,717,545) of points redeemed for rewards by the end of the study period. AYP from control zones gained 567,590 points with 62.2% (352,898) redeemed for rewards (Appendix).

### Coverage of key sexual and reproductive health services

Overall, 63.8% (9,493/14,872) of AYP accepting a PPC in the intervention arm accessed at least one key SRH service during the *whole* study period, compared to 5.4% (776/14,500) in the control arm (adjPR 12.3 95%CI 9.3–16.2, *p* < 0.001) (Table 4). Among adolescent girls 15-19-years, coverage was 72.1% (3,604/4,999) in the intervention arm compared to 5.5% (253/4,570) in the control arm (adjPR 13.8, 95%CI 10.2–18.6, *p* < 001) and among adolescent boys 15-19years, coverage was 68.3%(2,292/3,357) in the intervention arm compared to 6.7% (206/3,065) in the control arm (adjPR 10.6, 95%CI 8.2–13.8, *p* < 0.001). Results were similar among young women 20-24years (55.2%, 2,404/4,359) and young men 20-24years (55.3%, 1,193/2,157) in the intervention arm compared to the control arm (3.8%, 174/4,574; adjPR 16.0, 95%CI 11.1–22.9, *p* < 0.001 and 6.2% 143/2,291; adjPR 10.1, 95%CI 6.1–16.7, *p* < 001, respectively).

**Table 4:** Coverage of key Sexual and reproductive health services (HIV testing, condom collection, contraceptive collection, PrEP, VMMC, and ART), by trial arm

Uptake of at least 1 key SRH service	Coverage of SRH services in intervention arm			Coverage of services in control arm			*Adjusted PR	95% CI	*P* value
	*N*	*n*	%	*N*	*n*	%			
^1^ **Among those accepting PPC card as denominator, accessing services during the whole study period**									
Overall	14,872	9,493	**63.8%**	14,500	776	**5.4%**	12.3	9.3–16.2	< 0.001
15-19yo girls	4,999	3,604	**72.1%**	4,570	253	**5.5%**	13.8	10.2–18.6	< 0.001
20-24yo women	4,359	2,404	**55.2%**	4,574	174	**3.8%**	16.0	11.1–22.9	< 0.001
15-19yo boys	3,357	2,292	**68.3%**	3,065	206	**6.7%**	10.6	8.2–13.8	< 0.001
20-24yo men	2,157	1,193	**55.3%**	2,291	143	**6.2%**	10.1	6.1–16.7	< 0.001
^1^ **Among those accepting PPC card as denominator, accessing services during the last 12 months of the study period**									
Overall	14,872	6,545	44.0%	14,500	219	1.5%	30.3	22.3–41.1	< 0.001
15-19yo girls	4,999	2,481	49.6%	4,570	92	2.0%	26.0	18.7–36.2	< 0.001
20-24yo women**	4,359	1,718	39.4%	4,574	45	1.0%	47.2	26.8–83.0	< 0.001
15-19yo boys	3,357	1,531	45.6%	3,065	53	1.7%	31.2	20.0-48.6	< 0.001
20-24yo men	2,157	815	37.8%	2,291	29	1.3%	30.2	21.2–42.9	< 0.001
^1^ **Among those enumerated as denominator, accessing services during the whole study period**									
Overall	20,772	9,493	**45.7%**	20,093	776	**3.9%**	12.3	9.3–16.2	< 0.001
15-19yo girls	6,229	3,604	**57.9%**	5,778	253	**4.4%**	14.0	10.3–19.1	< 0.001
20-24yo women	5,624	2,404	**42.7%**	5,621	174	**3.1%**	15.1	10.7–21.3	< 0.001
15-19yo boys	4,944	2,292	**46.4%**	4,692	206	**4.4%**	11.1	8.3–14.8	< 0.001
20-24yo men	3,975	1,193	**30.0%**	4,002	143	**3.6%**	9.7	5.8–16.3	< 0.001
^1^ **Among those enumerated as denominator, accessing services during the last 12 months of the study period**									
Overall	20,772	6,545	31.5%	20,093	219	1.1%	30.2	22.5–40.6	< 0.001
15-19yo girls	6,229	2,481	39.8%	5,778	92	1.6%	26.5	19.0-36.8	< 0.001
20-24yo women**	5,624	1,718	30.5%	5,621	45	0.8%	44.6	25.9–76.6	< 0.001
15-19yo boys	4,944	1,531	31.0%	4,692	53	1.1%	32.4	20.7–50.5	< 0.001
20-24yo men	3,975	815	20.5%	4,002	29	0.7%	28.9	20.3–41.2	< 0.001

*adjusted for community

**Two zones that are outliers in control zones resulted in high PR as there was no access of services in this age group in the control zones

^1^ Age at time of consent to receive a Yathu Yathu prevention points card (PPC); PR = Prevalence ratio

Among AYP accepting a PPC, 44.0% (6,545/14,872) in the intervention arm accessed at least one key SRH service during the last *12 months* of the study period, compared to 1.5% (219/14.500) in the control arm (adjPR 30.3 95%CI 22.3–41.1, *p* < 0.001). Results were similar, in terms of the comparison of the intervention with the control arm, when all enumerated AYP, regardless of consent to accept a PPC, were included in the denominator (Table 4).

Among AYP accessing at least one key SRH service, HIV testing was the most accessed service in intervention (93.1% 8.841/9,493) and control arms (73.3%; 569/776). This was followed by collection of male and female condoms (49.5% 4,072/9,493 for intervention and 49.7% 387/776 for control).

Among AGYW who accessed at least one key SRH service, 14.4% (862/6008) accessed contraceptives in the intervention arm and 11.7% (50/427) in the control arm. Among ABYM who accessed at least one key SRH service, 0.9% (32/3485) accessed VMMC in the intervention arm and 4.0% (14/349) in the control arm. Using acceptance of a PPC as the denominator, HIV testing was the most accessed key SRH service in both arms, with 59.4% (8,841/14,872) in intervention arm compared to 3.9% (569/14,500) in the control arm (adjPR 15.6; 95%CI 11.5–21.0, *p* < 0.001) (Table 5).

**Table 5:** Coverage per key SRH service (HIV testing, condom collection, contraceptive collection, PrEP, VMMC and ART) and mean number of individual services accessed among AYP in the intervention arm and control arm among those that accepted a PPC during the whole study period

Uptake of key SRH service	Intervention arm				Control arm				^1^Adjusted PR	95% CI	*P* value
	*N*	*n*	%	*Mean number of services per participant	*N*	*n*	%	*Mean number of services per participant			
HIV testing	14,872	8,841	59.4%	1.13 (16, 769/14,872)	14,500	569	3.9%	0.044 (638/14,500)	15.6	11.5–21.0	< 0.001
Condom collection	14,872	4,702	31.6%	0.70 (10, 486/14,872)	14,500	386	2.7%	0.04 (595/14,500)	11.8	8.3–16.6	< 0.001
^2^Contraceptives collection	9,358	862	9.2%	2.01 (1, 886/9, 358)	9,144	50	0.5%	0.0007 (73/9,144)	19.4	12.4–30.3	< 0.001
^3^PrEP collection	14,459	6	0.0%	0.0007 (11/14,4,59)	14,500	4	0.0%	1.50 (6/4)	NA	NA	NA
^4^VMMC	5,514	32	0.6%	NA	5,356	14	0.3%	NA	1.7	1.0–3.0	0.048
^5^ART collection	323	50	15.5%	1.68 (84/50)	13	6	46.2%	2.00 (12/6)	NA	NA	NA

^1^ adjusted for community; ^2^ N = AGYW only; ^3^ N = AYP who tested HIV negative or did not report or test HIV positive; ^4^ N = ABYM only reporting not circumcised; ^5^ N = AYP that tested HIV positive or self reported as HIV positive

*numerator = total number of services accessed during whole study period, denominator = N, accepted a PPC

PR = Prevalence Ratio

## Discussion

Our community-based, peer-led, and incentivised intervention substantially increased the coverage and uptake of SRH services among AYP. Among AYP who accepted a PPC in the intervention zones, more than two thirds accessed at least one key SRH service from the hubs compared to less than 6% from the control zones accessing services at the health facility. AYP in intervention zones were 10–16 times more likely to access key SRH services and services were accessed more frequently by AYP residing in intervention zones than those in control zones. The implementation of the intervention achieved high fidelity, as evidenced by high staff retention, service provision on most planned service days and all but one of the core services delivered.

In our study, we are not able to disentangle the relative contribution of each of the three components (peer-led community-based spaces, PPC system and community engagement) to the impact of the intervention. The components were offered as a comprehensive intervention package, and we believe each component is important to achieve successful delivery of SRH to AYP. The establishment of walk-in community hubs away from the health facility and closer to homes, staffed by young people offering a comprehensive package of SRH services, addressed some barriers young people experience to engage in SRH [20]. In the formative phase of the study, they stated that fear of disapproval by health care workers prevented them from accessing SRH services at the health facility [17]. The role of community engagement, especially with parents and older people, in overcoming cultural norms associated with AYPs accessing SRH services appeared to be important in the success of the intervention [21]. This is corroborated by findings showing the importance of community engagement to encourage AYP participation in interventions in Zambia and Tanzania, especially with respect to cultural norms that hinder condom use and other contraceptive use among AYP [21–23]. Lastly, AYP from intervention and control zones were “rewarded” for accessing SRH services by receiving prevention points (incentives) to “nudge” or encourage uptake of services [13]. The observation that most AYP from intervention zones redeemed their accumulated points for rewards by the end of the study period, shows this may have been a motivating factor to access services. This is consistent with a similar intervention using incentives conducted in South Africa, which showed that users that accessed incentives were more likely to have an HIV test at the testing centres [24]. However, as control participants were also able to gain points and rewards, our data suggest incentivising access to SRH services alone is insufficient to overcome barriers AYP experience in accessing the health facility.

While the intervention had a substantial impact on coverage and uptake of SRH services supporting evidence of acceptability of a community-based intervention and consistent with other community-based studies such as the P-ART-Y study [11], not all age and sex groups were reached equally. During enumeration, ABYM were less likely to be found at home; although when they were home, acceptance of PPC was similar to AGYW. Young men aged 20–24 or those that were reported to be married were less likely to be home and so were not provided with cards. This unavailability of young men at home is consistent with other studies such as the HPTN 071 (PopART) community-randomised trial (of which the P-ART-Y study was a sub-study) with the intervention package delivered through door-to-door services [25, 26] However, among those accepting a card, two thirds of young men aged 20–24 accessed services at the hubs. In addition, although young men did not access services as often as women, the proportion accessing HTS was similar across both sexes implying that the hubs are acceptable for both sexes to access services. This is contrary to a systematic review that reported out-of-facility services were dominated by young men [9].

Among AYP in intervention zones who accepted a PPC almost three quarters accessed at least one service with over two thirds of these accessing services more than once. Uptake was highest among adolescent girls and boys aged 15-19-years, largely driven by uptake of HIV testing, perhaps indicating higher preference for hubs, and so lack of other accessible places, among the younger age groups compared to the older age groups. Reaching this age-group where 39%-51% report never having tested for HIV, compared to 7%-20% among the 20-24year old age groups [27], may be key in ensuring gains made in reducing new HIV infections are maintained. AYP who lived in households with more than 4 members were more likely to access services. This may indicate an increased need or desire for rewards, such as soap and toothbrushes, which in larger households may not be a priority purchase in comparison to other needs such as food. However, it is possible that in such households, there may have been other AYP that accessed hubs potentially resulting in positive peer pressure; this has been noted especially for young men in encouraging condom use in a study conducted in Lusaka among AYP [28] and in findings from the HPTN 071 study which found that having another adult in a household who had tested for HIV was associated with men testing for HIV [29].

While the intervention achieved high fidelity, this was negatively impacted due to COVID-19 and periods of unrest across the country resulting in closures and stock-outs of products such as oral contraceptives and HIVST kits at the hubs and local health facilities, with some due to the impact of COVID-19 on supply chain systems worldwide. This potentially impacted uptake of these services. Stock-outs at public health facilities have been reported to negatively impact uptake of SRH services by healthcare workers including in Zambia [30]. Clearly, a robust procurement system is essential to avoid stock outs.

While uptake of condoms and contraceptives were higher in the intervention arm compared to the control arm, it was still not as expected. This could have been influenced by persisting negative cultural norms despite community engagement, although, for condoms, collection may have been underreported as collection could be anonymous. However, the least accessed services were PrEP and PEP initiation, and VMMC, which were not provided at the hubs and required referral to the health facility. This may point to weaknesses in the referral system, despite having a desk at the clinic to guide AYP referred to the facility, to continued barriers in accessing SRH services, such as negative staff attitudes, at the health facility despite having youth-friendly corners [23] and low knowledge of PEP and PrEP.

### Strengths and limitations

A strength of our research is the rigorous design of the study adding to existing evidence for community based SRH service delivery. Another strength is that we were able to use the PPC to measure service use in real time, in both study arms. These data allowed us to measure various implementation domains more accurately, for example dose-received, compared to self-report. The high uptake of the PPC indicates the likely feasibility of using a similar card system in other interventions.

However, there are some potential limitations, including the effect that COVID-19 may have had on our study findings. While Yathu Yathu hubs were closed for 3 months during COVID-19, health facility attendance may have also decreased thus affecting the difference in coverage. AYP in the control arm may have presented their cards less than those in the intervention arm and we do not have data for those AYP without cards who may have accessed services at the health facility.

## Conclusion

This peer-led community-based SRH intervention achieved high coverage of SRH services, driven by a high uptake of HIV testing services and likely achieved through the provision of youth-friendly SRH service provision away from the health facility. It shows that provision of incentivised SRH services by peers in the community is feasible and effective in increasing access to HIV testing services, particularly for adolescents aged 15–19 years. Programmes providing, or considering providing, community-based service delivery, would strengthen service delivery by including community engagement with parents/guardians to facilitate community-wide acceptance of AYP accessing SRH services, and ensuring AYP are meaningfully engaged to ensure their needs are being met. Future research should include addition of on-site provision of PrEP, PEP, VMMC and contraceptives such as IUD/Implant to increase uptake as AYP were referred to the health facility to access these services, with low uptake.

### Electronic supplementary material

Below is the link to the electronic supplementary material.

Supplementary Material 1

## Data Availability

The datasets analysed for the current analysis are available from the corresponding author on reasonable request.

## Abbreviations

ABYM: Adolescent Boys and Young Men
AGYW: Adolescent Girls and Young Women
AYP: Adolescents and Young People
ART: Anti-Retroviral Therapy
CAB: Community Advisory Board
aCAB: Adolescent Community Advisory Board
COVID-19: Sars-Cov-2 Virus
CSE: Comprehensive Sexuality Education
CRT: Cluster Randomised Trial
HIV: Human Immunodeficiency Virus
HPTN 071: HIV Prevention Trials Network 071
HTS: HIV Testing Services
OR: Odds Ratio
PEP: Post Exposure Prophylaxis
PopART: Population Effects of Antiretroviral Therapy to reduce HIV transmission
PrEP: Pre-Exposure Prophylaxis
PPC: Prevention Points Card
PR: Prevalence Ratio
PSW: Peer Support Worker
SRH: Sexual and Reproductive Health
STI: Sexually transmitted infection
TB: Tuberculosis
UNAIDS: Joint United Nations programme on HIV and AIDS
VMMC: Voluntary Medical Male Circumcision
ZAMPHIA: Zambia Population-based HIV/AIDS Impact Assessment
