# Supplementary material for: The impact of an innovative community-based peer-led intervention on uptake and coverage of sexual and reproductive health services among adolescents and young people 15–24 years old: results from the Yathu Yathu cluster randomised trial
Source: BMC Public Health. 2024 May 28;24:1424. doi: 10.1186/s12889-024-18894-z (PMC11134624; doi:10.1186/s12889-024-18894-z)
Supplement: Supplementary file 1 [file 12889_2024_18894_MOESM1_ESM.docx]

Supplementary Appendix

This Appendix has been provided by the authors to give readers additional information about the study results and their work.

Table 1: SRH services accessed per capita (dose-received) among AYP who accepted and used their card at least once in Intervention arm, by age and sex

**Intervention - Male**	**Total accepting PPC and use at least once**						**Total number of visits**	
**15-17**	**1,626**						**10,185**	
**18-19**	**992**						**5,022**	
**20-24**	**1,338**						**4,931**	
**Service**	**Number accessed 15-17**	**Per capita**	**Number accessed 18-19**	**Per capita**	**Number accessed 20-24**	**Per capita**	**Number accessed total**	**Per capita**
first hub visit (one-off)	1604	0.99	990	1.00	1330	0.99	3924	0.99
alcohol screening for males (one-off)	1590	0.98	975	0.98	1308	0.98	3873	0.98
previously tested for HIV(one-off)	1603	0.99	976	0.98	1298	0.97	3877	0.98
collection of male condoms (max. once a week)	1940	1.19	1231	1.24	1519	1.14	4690	1.19
entrepreneurship skills session (max. 4 per year	0	0.00	1	0.00	2	0.00	3	0.00
Comprehensive sexuality education	4951	3.04	2461	2.48	2144	1.60	9556	2.42
collection of self-test(s) (max. 2 tests, once in the 3 months)	23	0.01	41	0.04	68	0.05	132	0.03
return a self-test with a HIV-negative result	13	0.01	22	0.02	52	0.04	87	0.02
collection of female condoms (max. once a month)	20	0.01	12	0.01	17	0.01	49	0.01
male condom use demonstration (one-off)	765	0.47	541	0.55	708	0.53	2014	0.51
screened for STI	1690	1.04	1029	1.04	1280	0.96	3999	1.01
HIV-test done at the hub (max. 4 times a year)	2618	1.61	1398	1.41	1530	1.14	5546	1.40
HIV-test at the clinic (max. 4 times a year)	0	0.00	0	0.00	6	0.00	6	0.00
education and entertainment session	1469	0.90	672	0.68	632	0.47	2773	0.70
bring friend	597	0.37	273	0.28	265	0.20	1135	0.29
STI treatment started	5	0.00	4	0.00	11	0.01	20	0.01
collection of ART	4	0.00	3	0.00	7	0.01	14	0.00
collection of PrEP	0	0.00	1	0.00	1	0.00	2	0.00
alc. support	83	0.05	42	0.04	66	0.05	191	0.05
Accepted VMMC procedure (one-off)	17	0.01	8	0.01	7	0.01	32	0.01
return of a self-test kit with a positive result	1	0.00	3	0.00	1	0.00	5	0.00
initiation of ART (one-off)	0	0.00	0	0.00	3	0.00	3	0.00
initiation of PrEP (one-off)	0	0.00	1	0.00	1	0.00	2	0.00
initiation of PEP (one-off)	0	0.00	0	0.00	1	0.00	1	0.00

TB screening	2029	1.25	1143	1.15	1356	1.01	4528	1.14
sanitary cup (one off)	NA		NA		NA		NA	
covid face masks (one-off)	1198	0.74	719	0.72	960	0.72	2877	0.73
TB treatment started	0	0.00	0	0.00	0	0.00	0	0.00
**Total services**	**22272**		**12583**		**14612**		**49467**	
**Intervention-Female**	**Total accepting PPC and use at least once**						**Total number of visits**	
**15-17**	**2524**						**21349**	
**18-19**	**1575**						**10484**	
**20-24**	**2919**						**13550**	
**Service**	**Number accessed 15-17**	**Per capita**	**Number accessed 18-19**	**Per capita**	**Number accessed 20-24**	**Per capita**	**Number accessed total**	**Per capita**
first hub visit (one-off)	2512	1.00	1567	0.99	2909	1.00	6988	1.00
alcohol screening for females (one-off)	2491	0.99	1540	0.98	2852	0.98	6883	0.98
previously tested for HIV (one-off)	2494	0.99	1557	0.99	2847	0.98	6898	0.98
collection of male condoms (max. once a week)	1805	0.72	1260	0.80	2175	0.75	5240	0.75
sanitary pad	6829	2.71	3321	2.11	4643	1.59	14793	2.11
entrepreneurship skills session (max. 4 per year	1	0.00	0	0.00	1	0.00	2	0.00
comprehensive sexuality education	9626	3.81	4457	2.83	4750	1.63	18833	2.68
collection of self-test(s) (max. 2 tests, once in the 3 months)	162	0.06	138	0.09	267	0.09	567	0.08
return a self-test with a HIV-negative result	114	0.05	96	0.06	178	0.06	388	0.06
collection of female condoms (max. once a month)	209	0.08	121	0.08	173	0.06	503	0.07
male condom use demonstration (one-off)	1209	0.48	768	0.49	1383	0.47	3360	0.48
screened for STI	3105	1.23	1861	1.18	3078	1.05	8044	1.15
refill contraceptives (max. once in 28 days)	144	0.06	285	0.18	595	0.20	1024	0.15
ANC attendence (max. 4 visits per pregnancy)	11	0.00	17	0.01	21	0.01	49	0.01
sign up for ANC (once per year)	11	0.00	11	0.01	33	0.01	55	0.01
HIV-test done at the hub (max. 4 times a year)	4869	1.93	2552	1.62	3699	1.27	11120	1.58
HIV-test at the clinic (max. 4 times a year)	3	0.00	1	0.00	3	0.00	7	0.00
start of oral or injectable contraceptives (one-off)	159	0.06	211	0.13	492	0.17	862	0.12
education and entertainment session	3064	1.21	1395	0.89	1601	0.55	6060	0.86
join a mobile support group (HIV-positive, one-off)	2	0.00	1	0.00	4	0.00	7	0.00
bring friend	903	0.36	567	0.36	680	0.23	2150	0.31
STI treatment started	6	0.00	18	0.01	16	0.01	40	0.01

collection of ART	13	0.01	7	0.00	29	0.01	49	0.01
collection of PrEP	2	0.00	1	0.00	1	0.00	4	0.00
alc. support	163	0.06	118	0.07	139	0.05	420	0.06
return of a self-test kit with a positive result	4	0.00	6	0.00	5	0.00	15	0.00
initiation of ART (one-off)	3	0.00	3	0.00	12	0.00	18	0.00
initiation of PrEP (one-off)	2	0.00	1	0.00	0	0.00	3	0.00
initiation of PEP (one-off)	3	0.00	1	0.00	3	0.00	7	0.00
TB screening	3615	1.43	1971	1.25	3229	1.11	8815	1.26
collection of emergency contraceptives	19	0.01	21	0.01	45	0.02	85	0.01
sanitary cup (one off)	158	0.06	144	0.09	240	0.08	542	0.08
covid face masks (one-off)	1919	0.76	1179	0.75	2195	0.75	5293	0.75
TB treatment started	0	0.00	0	0.00	0	0.00	0	0.00
**Total services**	**45742**		**25254**		**38393**		**109389**	

Table 2: **Per capita of SRH services accessed (dose-received) among AYP that accepted and used their card at least once in control arm, by age and gender**

**Control-Male**	**Total accepting PPC and use at least once**						**Total number of visits**	
**15-17**	**216**						**432**	
**18-19**	**155**						**344**	
**20-24**	**197**						**371**	
**Service**	**Number accessed 15-17**	**Per capita**	**Number accessed 18-19**	**Per capita**	**Number accessed 20-24**	**Per capita**	**Number accessed total**	**Per capita**
first hub visit (one-off)	19	0.09	14	0.09	13	0.07	46	0.08
alcohol screening for males (one-off)	17	0.08	14	0.09	13	0.07	44	0.08
previously tested for HIV (one-off)	65	0.30	56	0.36	66	0.34	187	0.33
collection of male condoms (max. once a week)	103	0.48	96	0.62	135	0.69	334	0.59
entrepreneurship skills session (max. 4 per year	0	0.00	0	0.00	0	0.00	0	0.00
comprehensive sexuality education	228	1.06	159	1.03	115	0.58	502	0.88
collection of self-test(s) (max. 2 tests, once in the 3 months)	0	0.00	0	0.00	0	0.00	0	0.00
return a self-test with a HIV-negative result	0	0.00	0	0.00	0	0.00	0	0.00
collection of female condoms (max. once a month)	0	0.00	0	0.00	0	0.00	0	0.00
male condom use demonstration (one-off)	4	0.02	1	0.01	1	0.01	6	0.01
screened for STI	39	0.18	43	0.28	52	0.26	134	0.24
HIV-test done at the hub (max. 4 times a year)	10	0.05	6	0.04	7	0.04	23	0.04
HIV-test at the clinic (max. 4 times a year)	46	0.21	73	0.47	92	0.47	211	0.37
education and entertainment session	3	0.01	0	0.00	0	0.00	3	0.01
bring friend	35	0.16	22	0.14	22	0.11	79	0.14
STI treatment started	0	0.00	3	0.02	7	0.04	10	0.02
collection of ART	0	0.00	0	0.00	4	0.02	4	0.01
collection of PrEP	0	0.00	0	0.00	2	0.01	2	0.00
alc. support	0	0.00	0	0.00	0	0.00	0	0.00
accepted VMMC procedure (one-off)	7	0.03	2	0.01	5	0.03	14	0.02
return of a self-test kit with a positive result	0	0.00	0	0.00	0	0.00	0	0.00
initiation of ART (one-off)	0	0.00	0	0.00	0	0.00	0	0.00
initiation of PrEP (one-off)	0	0.00	0	0.00	2	0.01	2	0.00
initiation of PEP (one-off)	1	0.00	0	0.00	0	0.00	1	0.00

TB screening	57	0.26	40	0.26	45	0.23	142	0.25
covid face masks (one-off)	7	0.03	2	0.01	5	0.03	14	0.02
TB treatment started	0	0.00	0	0.00	0	0.00	0	0.00
**Total services**	**642**		**534**		**590**		**1766**	

**Control - Female**	**Total accepting PPC and use at least once**						**Total number of visits**	
**15-17**	**448**						**1109**	
**18-19**	**290**						**728**	
**20-24**	**309**						**562**	
**Service**	**Number accessed 15-17**	**Per capita**	**Number accessed 18-19**	**Per capita**	**Number accessed 20-24**	**Per capita**	**Number accessed Total**	**Per capita**
first hub visit (one-off)	29	0.06	24	0.08	31	0.10	84	0.08
alcohol screening for females (one-off)	24	0.05	19	0.07	27	0.09	70	0.07
previously tested for HIV (one-off)	181	0.40	131	0.45	140	0.45	452	0.43
collection of male condoms (max. once a week)	65	0.15	92	0.32	101	0.33	258	0.25
sanitary pad	7	0.02	6	0.02	2	0.01	15	0.01
entrepreneurship skills session (max. 4 per year	0	0.00	0	0.00	0	0.00	0	0.00
comprehensive sexuality education	645	1.44	336	1.16	148	0.48	1129	1.08
collection of self-test(s) (max. 2 tests, once in the 3 months)	0	0.00	0	0.00	2	0.01	2	0.00
return a self-test with a HIV-negative result	0	0.00	0	0.00	3	0.01	3	0.00
collection of female condoms (max. once a month)	0	0.00	2	0.01	0	0.00	2	0.00
male condom use demonstration (one-off)	1	0.00	0	0.00	4	0.01	5	0.00
screened for STI	128	0.29	106	0.37	127	0.41	361	0.34
refill contraceptives (max. once in 28 days)	3	0.01	13	0.04	21	0.07	37	0.04
ANC attendence (max. 4 visits per pregnancy)	4	0.01	9	0.03	17	0.06	30	0.03
sign up for ANC (once per year)	4	0.01	5	0.02	8	0.03	17	0.02
HIV-test done at the hub (max. 4 times a year)	12	0.03	13	0.04	8	0.03	33	0.03
HIV-test at the clinic (max. 4 times a year)	100	0.22	120	0.41	146	0.47	366	0.35
start of oral or injectable contraceptives (one-off)	4	0.01	8	0.03	24	0.08	36	0.03
education and entertainment session	4	0.01	3	0.01	1	0.00	8	0.01
join a mobile support group (hiv-positive, one-off)	0	0.00	0	0.00	0	0.00	0	0.00
bring friend	110	0.25	61	0.21	28	0.09	199	0.19
STI treatment started	8	0.02	6	0.02	11	0.04	25	0.02
collection of ART	0	0.00	2	0.01	3	0.01	5	0.00

collection of PrEP	0	0.00	0	0.00	0	0.00	0	0.00
alc. support	1	0.00	1	0.00	0	0.00	2	0.00
return of a self-test kit with a positive result	0	0.00	0	0.00	0	0.00	0	0.00
initiation of ART (one-off)	0	0.00	1	0.00	2	0.01	3	0.00
initiation of PrEP (one-off)	0	0.00	1	0.00	1	0.00	2	0.00
initiation of PEP(one-off)	0	0.00	0	0.00	0	0.00	0	0.00
TB screening	146	0.33	107	0.37	105	0.34	358	0.34
collection of emergency contraceptives	0	0.00	0	0.00	0	0.00	0	0.00
sanitary cup (one off)	0	0.00	0	0.00	0	0.00	0	0.00
covid face masks (one-off)	10	0.02	10	0.03	8	0.03	28	0.03
TB treatment started	0	0.00	0	0.00	1	0.00	1	0.00
**Total services**	**1487**		**1081**		**975**		**3543**	

Figure 1: Points gained and redeemed in intervention arm by age and gender

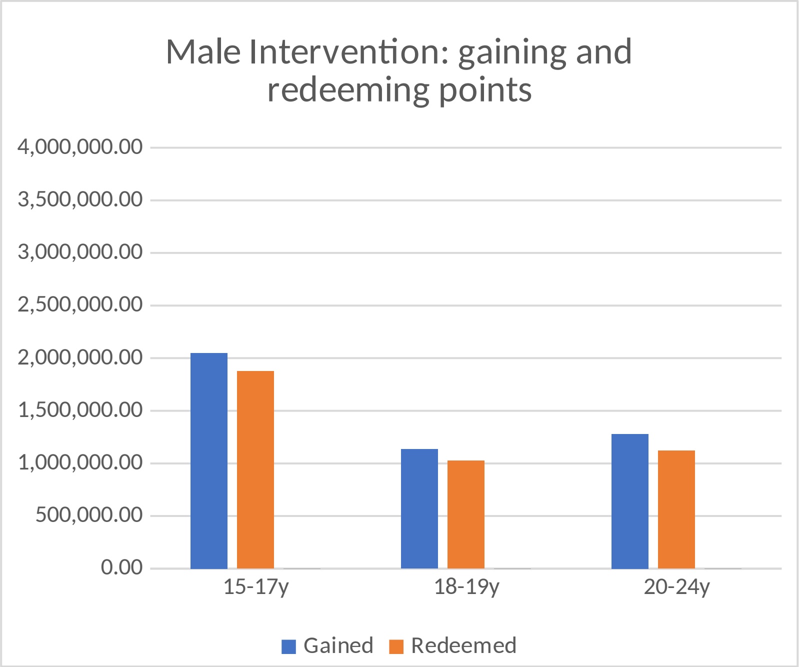

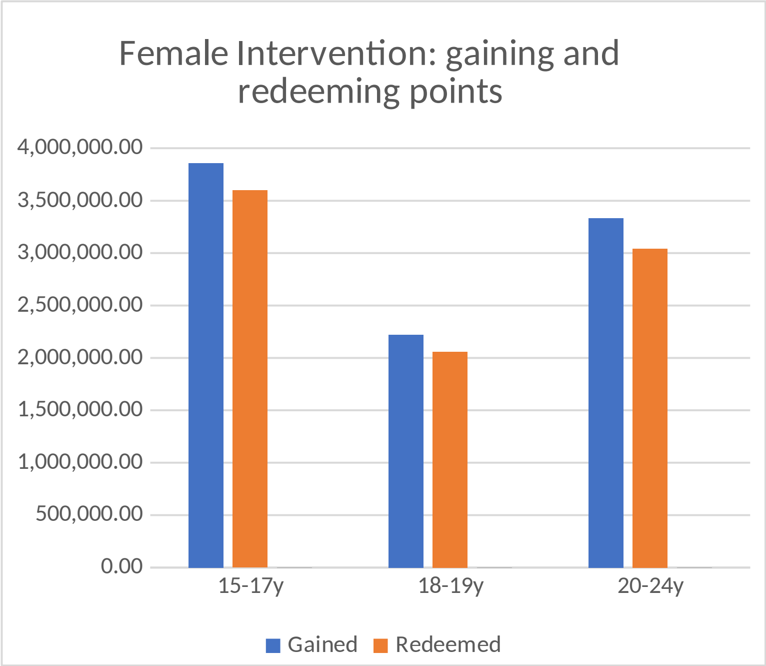

Figure 2: Points gained and redeemed in control arm by age and gender

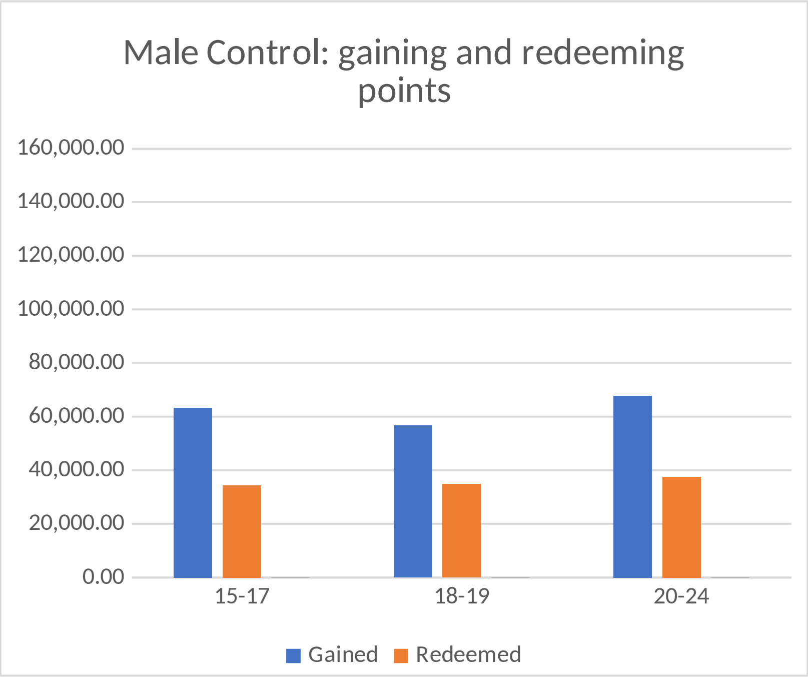

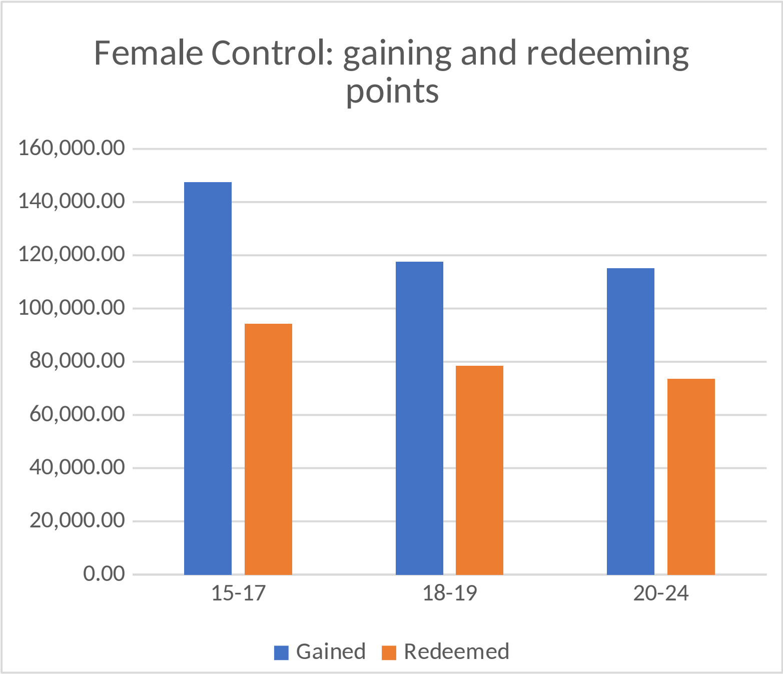

Table 3: Coverage of key Sexual and reproductive health services (HIV testing, condom collection, contraceptive collection, PrEP, VMMC, and ART), by service uptake and by trial arm

	Intervention arm			Control arm					
Uptake of key SRH service	N	n	%	N	n	%	[1]Adjusted PR	95% CI	P value
Among those accepting PPC accessing services during the whole study period									
One or more key services	14,872	9,493	63.8%	14,500	776	5.4%	12.3	9.3-16.2	<0.001
HIV-testing only	14,872	8,841	59.4%	14,500	569	3.9%	15.6	11.5-21.0	<0.001
Condom collection only	14,872	4,702	31.6%	14,500	386	2.7%	11.8	8.3-16.6	<0.001
[2]Contraceptives only	9,358	862	9.2%	9,144	50	0.5%	19.4	12.4-30.3	<0.001
[3]PrEP only	14,459	6	0.0%	14,500	4	0.0%	NA	NA	NA
[4]VMMC only	5,514	32	0.6%	5,356	14	0.3%	1.7	1.0-3.0	0.048
[5]ARTonly	323	50	15.5%	13	6	46.2%	NA	NA	NA
Among those accepting PPC, accessing services the last 12 months of the study period									
Overall	14,872	6,545	44.0%	14,500	219	1.5%	30.3	22.3-41.1	<0.001
HIV-testing only	14,872	5,787	38.9%	14,500	148	1.0%	39.4	29.5-52.5	<0.001
Condom collection only	14,872	3,825	25.7%	14,500	117	0.8%	31.3	20.7-47.3	<0.001
Contraceptives only	9,358	688	7.4%	9,144	19	0.2%	35.3	20.9-59.6	<0.001
Among those enumerated, accessing services during the whole study period									
Overall	20,772	9,493	45.7%	20,093	776	3.9%	12.3	9.3-16.2	<0.001
HIV-testing only	20,772	8,841	42.6%	20,093	569	2.8%	15.6	11.5-21.1	<0.001
Condom collection only	20,772	4,702	22.6%	20,093	386	1.9%	11.7	8.6-16.0	<0.001
Contraceptives only	11,853	862	7.3%	11,399	50	0.4%	18.9	11.7-30.4	<0.001
Among those enumerated, accessing services during the last 12 months of the study period									
Overall	20,772	6,545	31.5%	20,093	219	1.1%	30.2	22.5-40.6	<0.001
HIV-testing only	20,772	5,787	27.9%	20,093	148	0.7%	39.4	29.7-52.2	<0.001
Condom collection only	20,772	3,825	18.4%	20,093	117	0.6%	31.2	21.4-45.6	<0.001
Contraceptives only	11,853	688	5.8%	11,399	19	0.2%	34.4	19.9-59.4	<0.001

[1] adjusted for community; [2] N= AGYW only ; [3] N=AYP who tested HIV negative or did not report or test HIV positive;[4] N=ABYM only ; [5] N= AYP that tested HIV positive or self reported as HIV positive

TIDieR _ View generated form.pdf
